# Biological invasions increase the richness of arbuscular mycorrhizal fungi from a Hawaiian subtropical ecosystem

**DOI:** 10.1007/s10530-018-1710-7

**Published:** 2018-03-21

**Authors:** Sofia I. F. Gomes, Vincent S. F. T. Merckx, Nicole A. Hynson

**Affiliations:** 1 Understanding Evolution Group, Naturalis Biodiversity Center, 2332 AA Leiden, The Netherlands; 2 Department of Botany, University of Hawaii Manoa, 3190 Maile Way Room 101, Honolulu, HI 96822 USA

**Keywords:** Arbuscular mycorrhizal fungi, Biogeography, Hawaiian Islands, Invasive trees, Biodiversity

## Abstract

Biological invasions can have various impacts on the diversity of important microbial mutualists such as mycorrhizal fungi, but few studies have tested whether the effects of invasions on mycorrhizal diversity are consistent across spatial gradients. Furthermore, few of these studies have taken place in tropical ecosystems that experience an inordinate rate of invasions into native habitats. Here, we examined the effects of plant invasions dominated by non-native tree species on the diversity of arbuscular mycorrhizal (AM) fungi in Hawaii. To test the hypothesis that invasions result in consistent changes in AM fungal diversity across spatial gradients relative to native forest habitats, we sampled soil in paired native and invaded sites from three watersheds and used amplicon sequencing to characterize AM fungal communities. Whether our analyses considered phylogenetic relatedness or not, we found that invasions consistently increased the richness of AM fungi. However, AM fungal species composition was not related to invasion status of the vegetation nor local environment, but stratified by watershed. Our results suggest that while invasions can lead to an overall increase in the diversity of microbial mutualists, the effects of plant host identity or geographic structuring potentially outweigh those of invasive species in determining the community membership of AM fungi. Thus, host specificity and spatial factors such as dispersal need to be taken into consideration when examining the effects of biological invasions on symbiotic microbes.

**Electronic supplementary material:**

The online version of this article (10.1007/s10530-018-1710-7) contains supplementary material, which is available to authorized users.

## Introduction

A large number of studies have examined the direct effects of biological invasions on the diversity of invaded communities (Vitousek et al. 1996; Sax and Gaines 2008; Vilà et al. 2011). However, it remains to be seen whether invasion effects aboveground are mirrored in belowground soil microbial communities or how microbial mutualists, such as mycorrhizal fungi, may reduce or exacerbate the negative effects of invasive plant species on native flora (Traveset and Richardson 2006; Desprez-Loustau et al. 2007; Pringle et al. 2009; Lekberg et al. 2013; Zubek et al. 2016). Because mutualisms underlie ecosystem functioning, productivity and stability (Klironomos et al. 2000; Renker et al. 2004; van der Putten et al. 2009; Wagg et al. 2011) and can strongly influence plant invasions (Simberloff and Von Holle 1999; Richardson et al. 2000; Lekberg et al. 2013), the impact of biological invasions on mutualist communities is of both theoretical and practical importance.

In circumstances where invaders compete directly with native organisms, most studies and meta-analyses support a loss of local diversity as a direct result of invasions (Fridley et al. 2007; Vilà et al. 2011; Chase et al. 2015). However, rather than invaders having direct effects on mutualists, the effects of invasions on mutualists may be indirect for example, via changes in the density or abundance of host organisms (Simberloff and Von Holle 1999). Consequently, conceptual frameworks for invasion processes and outcomes based on the direct interactions of invasive species with their native counterparts cannot necessarily be assumed to apply to mutualistic organisms (Richardson et al. 2000; Callaway et al. 2004).

The symbiosis between plants and mycorrhizal fungi is one of the most widespread mutualisms on earth (Smith and Read 2008). The most common type of mycorrhizal fungi are the arbuscular mycorrhizal fungi (subphylum Glomeromycotina, former phylum Glomeromycota, Spatafora et al. 2016) which form obligate associations with > 80% of terrestrial plant species, including many invasive plant species (Brundrett 2009; Pringle et al. 2009). In this mutualism, the host plant passes carbon fixed through photosynthesis on to its associated AM fungi in exchange for increased access to growth-limiting soil nutrients, especially phosphorus (Smith and Read 2008). Due to the importance of AM fungi for host plant fitness and their broad associations with a diversity of hosts, they are ideal candidates to examine the effects of invasions on microbial mutualists’ community dynamics. In general, the arbuscular mycorrhizal symbiosis is thought to be relatively non-specific where host plants can benefit from a diversity of geographically or phylogenetically disparate AM fungi and vice versa (Moora et al. 2011; Davison et al. 2015; Lekberg and Waller 2016; but see van der Heijden et al. 1998; Vandenkoornhuyse et al. 2003; Alguacil et al. 2009; Bunn et al. 2015). However, symbiont compatibility is only one of many ecological filters that AM fungi must pass through in order to establish. Other factors such as dispersal ability, environmental suitability, and intra-guild biotic interactions may outweigh the relative importance of invasive host compatibility on the ability of AM fungi to establish and persist (Leibold et al. 2004; Filotas et al. 2010; Pillai et al. 2014).

Various scenarios have been put forth as to how plant invasions may alter the diversity of mycorrhizal fungi. Previous research has suggested that invasive plants may have positive (Lekberg et al. 2013; Chen et al. 2015), neutral (Richardson et al. 2000; Wolfe et al. 2010), or negative (Mummey and Rillig 2006; Murat et al. 2008) effects on the species richness of mycorrhizal fungi (see Dickie et al. 2017 for a recent review). A decrease in species richness may be the result of introducing non-mycorrhizal hosts or invaders that require fewer fungal partners than native hosts (Richardson et al. 2000; Pringle et al. 2009; Nuñez and Dickie 2014), while no change in species richness may be due to invasive host plants partnering with the extant mycorrhizal community (Richardson et al. 2000; Catford et al. 2009; Pringle et al. 2009; Moora et al. 2011; Nuñez and Dickie 2014), and increases in richness may be the result of co-invasions of non-native hosts and fungi (Dickie et al. 2010; Lekberg et al. 2013; Bogar et al. 2015), yielding net impacts of plant invasions on mycorrhizal fungal diversity that are difficult to predict (Dickie et al. 2017).

Previous studies of the effects of plant invasions on AM fungal communities are primarily focused on forbs, grass and shrub invasions (Bunn et al. 2015). However, woody species such as trees are also common invaders, especially in the tropics, and the majority of invasive trees also associate with AM fungi (Nuñez and Dickie 2014). Prior research on AM fungi and invasions has also been focused on habitats in temperate or Mediterranean climates despite the fact that tropical islands tend to experience a disproportionate rate of plant invasions (Kueffer et al. 2010). To date there have been no studies that examine the effects of tree invasions on AM fungal diversity in tropical island ecosystems. To fill this gap, we explore the effects of tree-dominated invasions on AM fungal diversity on the island of Oahu in the Hawaiian archipelago, which is one of the invasion capitals of the world (Vitousek et al. 1996). There, three of the most common and pernicious invasive trees are strawberry guava (*Psidium cattleianum* Myrtaceae), Christmasberry (*Schinus terebinthifolius* Anacardiaceae), and Australian redcedar (*Toona ciliata* Meliacae). The first two were introduced to Hawaii between the early nineteenth century and sometime before 1911. Strawberry guava and Christmasberry are spread by birds, and the former is also dispersed by non-native ungulates and rodents. Both trees now form thick monodominant stands that have replaced historically native vegetation (Motooka et al. 2003). Australian red cedar was potentially introduced to Hawaii as early as the mid-nineteenth century and later broadly planted as fast-growing timber species and is now considered invasive (Wagner et al. 1999).

We test the hypothesis that independent of geographic location or host identity these tree invasions have led to similar changes in AM fungal richness, species incidence and phylogenetic community structure. We compare AM fungal richness across spatial gradients in paired native and invaded watersheds. We assess the impacts of invasions on the diversity of AM fungi by comparing diversity measures based on species incidence, with phylogenetic distances metrics. We chose to assess changes in AM fungal richness, species incidence and phylogeny because there are no previous studies that have compared the effects of invasion on all three making it difficult to predict whether invasions will lead to changes in all, some, or none of the above.

## Materials and methods

### Soil sampling

We established a total of 18 plots in three watersheds, with three plots per watershed dominated by native forest vegetation, and three dominated by one of three common invasive tree species. We defined native and invasive plots as having > 90% canopy cover of native or invasive vegetation, respectively. Percent cover for native and invasive plots was estimated by eye by a single observer (Korhonen et al. 2006). We chose > 90% cover of invasive or native species, because many effects of invasions are nonlinear and density-dependent, and not readily detectable until 50% or more invasive cover is reached (Thiele et al. 2009). To our best knowledge, this is the first molecular-based AM fungi survey in Hawaii and therefore we selected typical native and invasive vegetation types.

Native plots consisted of Hawaiian mesic lowland forest or montane rainforest with diverse canopies and understories dominated by the common native tree species *Metrosideros polymorpha* and *Acacia koa* in all three watersheds. Other plant species varied by watershed. The Palikea watershed had a mix of *Antidesma platyphyllum*, *Kadua affinis*, *Alyxia stellata*, *Cheirodendron trigynum*, *Ilex anomala*, *Melicope clusiifolia* and *Psychotria mariniana*. The Pahole/Kahanhaiki watershed had *Bobea elatior*, *Pouteria sandwicensis*, *Antidesma platyphyllum* and *Psychotria mariniana*, while the Manuwai watershed had *Diospyros sandwicensis* and *D. hillebrandii*, *Pisonia brunoniana* and *Nestegis sandwicensis*. While historically unmanaged, the native plots were generally within areas that were fenced in the last two decades to prevent disturbances to native vegetation caused by non-native ungulates. All native canopy species in this study have been reported to form arbuscular mycorrhizal associations (Koske et al. 1992). Invasive plots are classified as Hawaiian introduced mesic forest dominated by one of the invasive tree species *Psidium cattleianum*, *Schinus terebinthifolius* or *Toona ciliata,* and less abundant invasive trees such as *Grevillea robusta* and *Morella faya,* with minimal understories of invasive grass species such as *Megathyrsus maximus*. All of these species form arbuscular mycorrhizal associations (Brundrett 2009).

**Fig. 1:**
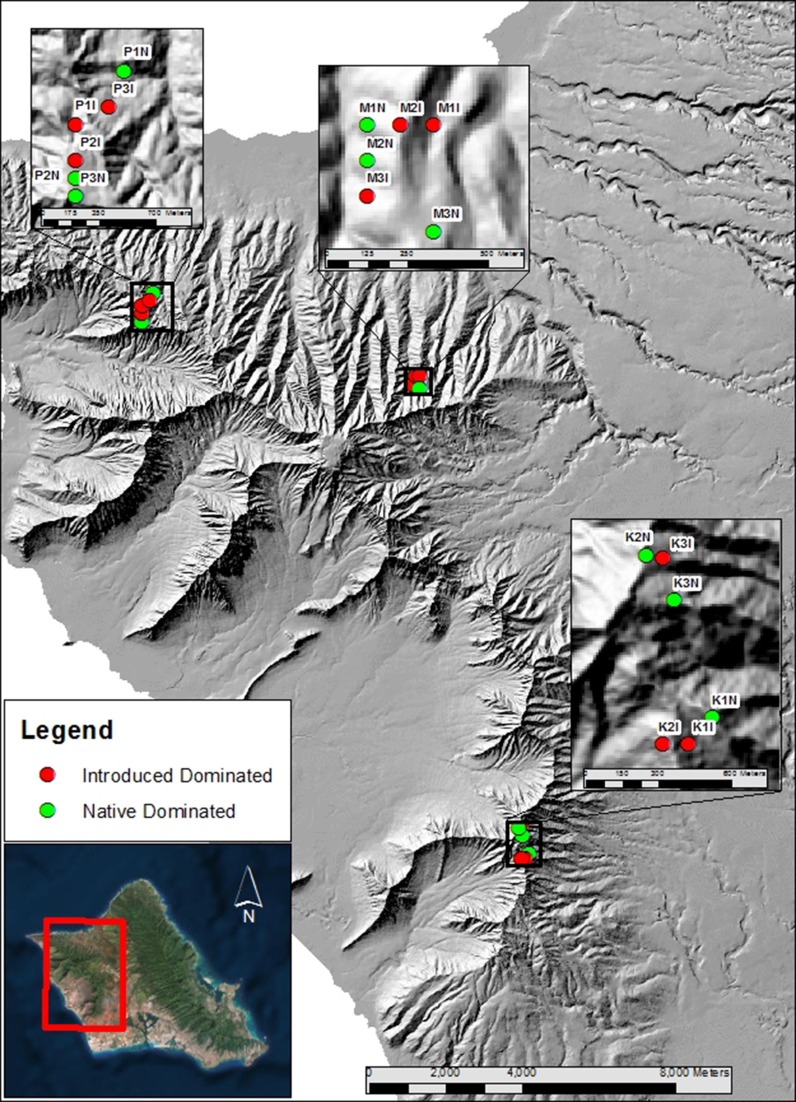
Map of Oahu island in Hawaii with sampling locations of 18 plots in three watersheds (K, Palikea; M, Manuwai; P, Pahole) dominated by native (green circles) and invasive (red circles) vegetation. Each plot was 24 m × 24 m. Within watershed, plots were separated by a minimum of 70 m, with a maximum separation of 780 m

Each of the 18 individual plots was 24 m × 24 m (576 m^2^). Within watersheds, plots were separated by a minimum of 70 m, with maximum separation of 780 m (Fig. 1; SI Table S1). Individual watersheds were separated by 7–17 km (Fig. 1). In each plot we established a regular grid with gridlines separated by 2 m and using a bulb planter sterilized between samples with 95% ethanol, sampled a single shallow (11 cm deep) soil core of approximately 430 mL inside each grid cell, yielding 144 soil cores per plot and 2592 soil cores in total (Fig. 1; SI Table S1). Within each plot we sampled an additional eight regularly spaced soil cores for soil chemical analysis. Each core was bagged individually, put on ice for transport back to the lab where within 8 h of sampling, we began drying soil cores in air-drying ovens at approximately 50 °C. Soil samples remained in dryers until fully dehydrated (3–5 days) and were then stored at room temperature pending processing (Cesaro et al. 2008; Janoušková et al. 2015; Tedersoo et al. 2014; Leon et al. 2016). Although previous studies (Bainard et al. 2010) have raised the concern of DNA degradation associated with drying the soil, Janoušková et al. (2015) showed that air-drying the soil up to 60 °C does not result in selective degradation of fungal DNA in the samples and this is a common practice for molecular studies of AM fungi (Cesaro et al. 2008; Tedersoo et al. 2014; Leon et al. 2016). Also, according to Janoušková et al. (2015), the amount of AM fungal DNA present in the dried samples was higher than in the frozen samples, therefore we chose this method of preservation for subsequent molecular analyses. We froze soil cores taken for chemical analysis at − 20 °C within the same time period. Samples were collected April–June 2014 with both invaded and native plots sampled throughout the entire range of dates. We recorded elevation, latitude and longitude for each plot using a handheld GPS (Garmin, Chicago, IL). Data on mean annual precipitation per plot was taken from the Hawaiian Rain Atlas (Giambelluca et al. 2012). Soil chemistry analysis was performed by the University of Hawaii’s College for Tropical Agriculture and Human Resources Agricultural Diagnostic Service Center.

### Molecular methods and bioinformatics

In total, for the 18 plots in the three watersheds, each of the 2592 dried soil cores were thoroughly homogenized using a sterilized mallet and then subsampled to 250 mg (± 10 mg) for DNA extraction. We extracted genomic DNA from each soil core following a CTAB extraction protocol modified to include 3% w/v polyvinylpyrrolidone and 2% v/v 2-mercaptoethanol in the lysis buffer. Initially, we considered examining within plot changes of AM fungal diversity under invasion. So, following this extraction, for each plot we pooled equal volumes from 4 to 44 DNA extracts of the 144 extracts total into six pools per plot (SI Figure S1), resulting in a total of 108 samples. However, while we sequenced each of these samples individually (see below) we ended up pooling samples by plot and analyzing our data with “plot” as the unit of replication (n = 9 per habitat type) to avoid issues of pseudoreplication and spatial autocorrelation of AM fungi. We purified DNA from 100 μL of pooled extract per plot using PowerClean Pro DNA Clean-up Kits (Mo Bio Laboratories, Carlsbad, CA).

For each of our samples we amplified a fragment of the nuclear ribosomal large subunit (LSU) following the nested PCR protocol of Kohout et al. (2014). Briefly, our first PCR used the forward primer LR1 and reverse primer FLR2 with cycling conditions followed Kohout et al. (2014), but omitting bovine serum albumin and using Q5 high-fidelity DNA polymerase (New England Biolabs, Ipswich, MA). We then cleaned PCR products using Sera-Mag Carboxylate-modified Magnetic beads (GE Healthcare, Pittsburgh, PA). Our second PCR used the primers 250f and FLR4 (Kohout et al. 2014). Following this second PCR, we performed another PCR clean-up, as before. We attached MiSeq flow cell adapters and 8 bp multiplex indices (Kozich et al. 2013) using a 15-cycle PCR with the following conditions: 95 °C for 55 s, then 15 × (95 °C for 30 s, 56 °C for 15 s, 72 °C for 30 s) with a final extension step of 72 °C for 600 s. We bead-cleaned amplicons as before, then used a Qubit2 spectrophotometer with dsDNA HS reagents (Life Technologies, Burlington, ON) to quantify DNA in each sample. We pooled the 108 samples in equimolar concentrations, then submitted samples for sequencing on an Illumina MiSeq platform using V3 600 cycle chemistry at the Hawaii Institute for Marine Biology. We split samples between two runs, each a random mixture of sites. The number of reads per library did not differ significantly between these two half-runs, and there was no significant difference in the number of invaded versus native-dominated pools in each run (Kruskal–Wallis tests; *P* values ≥ 0.30).

We pooled sequences from both half-runs, then we followed the UPARSE-based bioinformatics pipeline (Edgar 2013) in USEARCH v8.1.1861 (Edgar 2010) to process our sequences. We first merged the paired-end sequences when the forward and reverse read had at least 30 bases overlap. Reads were dereplicated, and OTUs (operational taxonomic units) were picked de-novo from these dereplicated reads. Reads were mapped to OTUs at 97% identity with de-novo chimera check. Ninety-seven percent is the recommended cut-off for use with the UPARSE pipeline (Edgar 2013) and is also commonly used with arbuscular mycorrhizal fungal large subunit data (Kohout et al. 2014; Lekberg et al. 2013). Following Lindahl et al. (2013), we discarded all the OTUs represented by less than five reads from each individual sample. Several studies have shown that read abundance from high throughput sequencing does not correlate with actual relative abundance among species (Amend et al. 2010; Nguyen et al. 2014). Therefore, we did not use sequence abundance per OTU in any of our downstream analysis; rather we just used OTU presence/absence. Raw sequences are deposited in the NCBI Short Read Archive under project number PRJNA312973. The OTUs obtained were queried against the NCBI database using the standard settings of the megaBLAST algorithm, and retained for further analysis the OTUs belonging to the Glomeromycotina sub-phylum (AM fungi).

### Assessing AM fungal diversity

We chose to compare measures of species incidence and phylogenetic diversity as previous work on plant invasions has shown that phylogeny and native species richness are useful predictors of the effects of invasion. For example, Strauss et al. (2006) found that the more complementary the phylogenies of native and invasive grass species are, the more resistance there is to invasion. Similarly, in a meta-analysis Levine et al. (2004) found support for the biotic resistance hypothesis where with greater native species richness, there is less likelihood of habitats becoming invaded. In addition, Bunn et al. (2014) showed that despite AM fungal species richness decreasing with increasing invasion density, community composition was not significantly altered.

Because observed species richness often under estimates true species richness in environmental microbial communities (Hughes et al. 2001) asymptotic estimates of AM fungi OTU richness were estimated based on the first three Hill numbers, namely species richness (*q* = 0), Shannon diversity (the exponential of Shannon entropy, *q* = 1), and Simpson diversity (the inverse Simpson concentration, *q* = 2) (Chao et al. 2014). Hill numbers offer numerous advantages over other diversity indices such as uniting species diversity and similarity, obeying the replication principle of species assemblages where samples (n) that have the same Hill number (X), but no overlap in community membership are represented by n × X, and they are expressed in units of effective number of species (Chao et al. 2014). To calculate Hill numbers, species rarefaction and extrapolation curves we used the R package *iNEXT* (Hsieh et al. 2016). These sample-size interpolation and extrapolation sampling curves are based on effective number of taxa under a nonparametric framework (Chiu and Chao 2016), and represent the diversity estimates for rarified and extrapolated samples in respect to the number of sampled plots, according to vegetation status (invasive or native). Comparisons of estimated and extrapolated OTU richness (*q* = 0), and diversity (*q* = 1, *q* = 2) in plots dominated by native versus invasive vegetation were evaluated by comparisons of mean OTU richness or diversity and the calculated 84% confidence intervals for each group. Chiu and Chao (2016) developed a bootstrap method to obtain unconditional variances and confidence intervals for the rarified and extrapolated estimators, with the purpose to compare richness and diversity of multiple assemblages. This unconditional variance assumes that the reference sample represents a random draw from a larger and unmeasured community, therefore the confidence intervals remain ‘open’ at the full-sample end of the curve, being more appropriate than the traditional variance estimators for inference of large species assemblages (Colwell et al. 2012). Differences among plots were considered statistically significant when their confidence intervals did not overlap. Payton et al. (2003) showed that for normally distributed confidence intervals, and asymmetric log-normal confidence intervals (MacGregor-Fors and Payton 2013), comparing 83–84% confidence intervals accurately mimics an α = 0.05, whereas 95% confidence intervals have a high probability of type I errors (Payton et al. 2003). Therefore, comparing means based on 95% confidence intervals results in a much more conservative α, closer to α = 0.01 (MacGregor-Fors and Payton 2013).

### AM fungi community statistics

To test whether invaded and native plots displayed different patterns of spatial separation or aggregation (which would confound our results), we used distance-based multivariate analysis of variance (Permanova with 1000 permutations), using the *Adonis* function in the R package *vegan* (Oksanen et al. 2017). For that, we calculated the Jaccard distance between all the plots based on the geographic coordinates (SI Table S1), using the *distm* function in the R package *geosphere* (Hijmans 2016), and tested if they were aggregated by status of the vegetation within watersheds (strata = watersheds).

To investigate the effect of invasion on AM fungi community composition we compared the composition of AM fungi between native and invasive plots. We used a univariate Permanova with Jaccard distances considering differences among watersheds (no strata) and within watersheds (strata = watersheds) to account for the nested design.

To test if individual OTUs were statistically associated with invasive or native vegetation plots, or whether there were OTUs predominantly associated with all the plots regardless of invasion status or watershed, we performed a species indicator analysis. We obtained the indicator species values with the *indicspecies* R package using the *multipatt* function (Cáceres and Legendre 2009), which tests for statistical significance of the highest association of species values of each group.

### Phylogenetic community structure

Because long rDNA sequences allow for robust phylogenetic analyses and species level resolution (Krüger et al. 2009), we added SSU–ITS–LSU reference sequences of identified AM fungi (Krüger et al. 2012) as backbone to our alignment to obtain more accurate relationships among the deeper clades. Sequences were aligned with MAFFT (Katoh 2013) and phylogenetic inference was performed with raxmlHPC-SSE3 (Stamatakis 2014) using the GTR + I + G model of substitution as determined by jModeltest v2.1.5 using the Akaike Information Criterion (AIC) (Darriba et al. 2012). Phylogenetic distances (sum of branch lengths) between the OTUs from the highest likelihood tree were used to calculate phylogenetic diversity. Phylogenetic beta diversity was measured among native and invasive dominated plots and among watersheds using *comdist* of the *picante* R package (Kembel et al. 2010), which calculates the mean pairwise distance inter-communities. Ordination methods were used to compare the dissimilarity matrices based on species composition and phylogenetic beta diversity among watersheds and considering invasion status. Additionally, we computed a Mantel test on these dissimilarity matrices to evaluate if the observed patterns were consistent, using the *mantel.test* function with 999 permutations in the *ape* R package. We compared the phylogenetic diversity of the AM fungal communities among native and invasive plots, and among watersheds considering total AM fungal diversity, and only particular clades, using a Permanova. In our study, we detected only few AM fungi belonging to the orders Paraglomerales, Archaeosporales and Diversisporales, as expected by using the selected LSU primers (Krüger et al. 2009). Therefore, we looked for differences in the phylogenetic diversity of AM fungi within the Glomerales among native and invasive plots, and among watersheds. There is no universal ideal cut-off value for species delimitation on AM fungi using the LSU region, and because using 97% OTU clustering may not reflect true species level resolution, we adopted a conservative approach of not over-splitting clades by only considering three well-supported clades within the order Glomerales for the subsequent analyses. We considered the Glomerales (clades I, II and III), and within this order, the Glomeraceae family (clades I and II), and finally the clades I and II individually.

### Soil chemistry and environmental data

Elevation, mean precipitation and soil chemistry were assessed for statistical association with watershed and invasion status of the vegetation. The normality test *shapiro.test* in R package *stats* (R Core Team 2016) revealed normality in pH values (*W* = 0.979, *P* = 0.936), calcium (*W* = 0.914, *P* = 0.101), magnesium (*W* = 0.940, *P* = 0.287) and nitrogen (*W* = 0.903, *P* = 0.064); and nonnormality in elevation (*W* = 0.898, *P* = 0.054), precipitation (*W* = 0.827, *P* = 0.004), phosphorous (*W* = 0.723, *P* = 0.0001) and potassium (*W* = 0.865, *P* = 0.015). Because some of the variables showed nonnormality, differences in environment among the three watersheds were assessed with a Kruskal–Wallis rank sum test, using the *kruskal.test* from the R package *stats* (R Core Team 2016) with Bonferroni corrections for multiple comparisons. The Mann–Whitney–Wilcoxon rank sum test was performed using the *wilcox.test* from the R package *stats* (R Core Team 2016) with Bonferroni corrections to assess differences in the environment between native and invasive plots. To examine the relationship between environmental variables and AM fungi community composition we used a partial redundancy analysis (RDA) on the presence/absence AM fungi operational taxonomic unit (OTU) contingency table.

## Results

### Sequencing results

Using a paired-end assembly approach, the initial 6,606,842 raw sequences were merged in a total of 2,162,226 paired-ended reads. Of these, 974,054 passed final quality control (excluding 110,143 putative chimeric sequences), and 766,293 (78.7%) were identified as belonging to the sub-phylum Glomeromycotina (and were therefore identified as AM fungi; Spatafora et al. 2016). High quality sequences were clustered in a total of 70 AM fungi OTUs at 97% similarity. The average identity of each OTUs to a fungal sequence in the GenBank database (including uncultured sequences) was 98.04% ± 1.92 (s.d.) in an alignment of 298.9 ± 8.69 bp. Only four OTUs (OTUs numbers: 13, 16, 37 and 49) matched with 100% identity to existing GenBank records, all from uncultured fungi present in the soil of agricultural fields in Japan (OTU 13) and Hungary (OTUs 16, 37 and 49).

In the 18 plots, the number of reads per plot was not correlated with the number of OTUs in that sampling unit (Pearson’s product-moment correlation, *r* = 0.316, *df* = 16, *P* = 0.202). The number of total reads in native plots did not differ significantly from the number of reads in invasive plots (Kruskal–Wallis test, Chi squared = 3.277, *df* = 1, *P* = 0.071). The same was observed for the total number of OTUs (Kruskal–Wallis test, Chi squared = 0.388, *df* = 1, *P* = 0.533).

### Estimates of AM fungal diversity in native and invasive plots

**Fig. 2:**
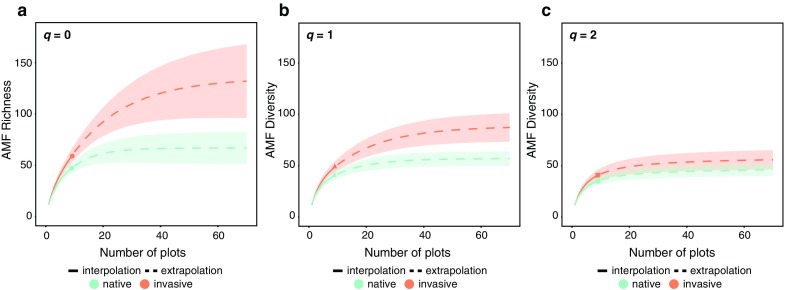
Sample-size- rarefaction and extrapolation sampling curves with confidence intervals for the plots with native (green) and invasive (red) dominant vegetation, according to the Hill numbers which includes species richness (**a**), Shannon diversity (the exponential of Shannon entropy, **b**), and Simpson diversity (the inverse Simpson concentration, **c**)

We found in total 46 and 59 AM fungi OTUs in the native and invasive plots, respectively which is an increase of approximately 22% overall. Of these, 11 were unique to native plots, while 24 were unique to the invasive plots, and 35 were shared among native and invasive plots. Rarefaction curves based on our observed data according to native or invasive status do not approach asymptote (Figure S2). However, the observed and extrapolated diversity measures (based on the Hill numbers *q* = 0, 1, 2; Fig. 2) showed that invaded sites had on average higher overall AM fungal richness than native ones and this pattern was consistent across plots and the three watersheds. Although the variance around the means for these estimators is considerable, the estimated AM fungal richness in invasive areas was consistently and significantly higher than in native ones based on 84% confidence for the Hill numbers *q* = 0 and 1 (Fig. 2a, b; note that confidence intervals do not overlap indicating that they are statistically significantly different). Yet, for *q* = 2 while average AM fungal diversity was higher in the invasive plots, the lower and upper 84% confidence intervals from invasive and native plots overlap, respectively (Fig. 2c).

**Fig. 3:**
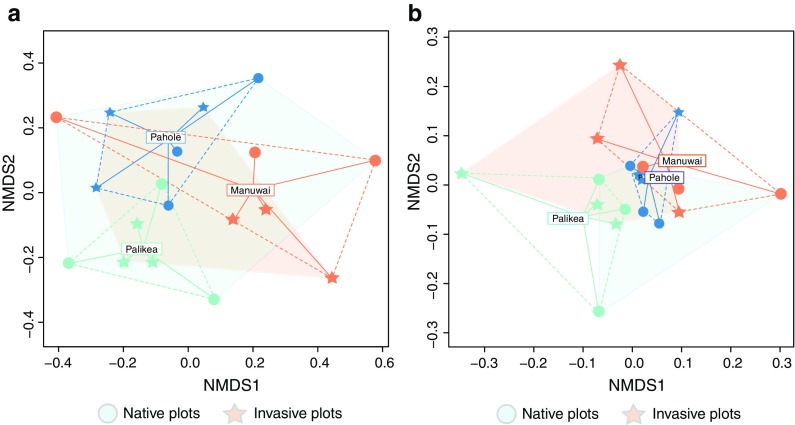
Nonmetric multidimensional scaling (NMDS) plots of AM fungal communities detected in the three watersheds: Palikea, green; Manuwai, orange; Pahole, blue. Native and invasive dominated plots are represented with circles and stars, respectively. The fungal community distances were calculated with **a** jaccard index (stress: 0.173); and **b** phylogenetic distances using *comdist* (stress: 0.177)

We observed no significant difference in spatial aggregation of plots by native and invasive status within watersheds (Permanova, *Pseudo*-*F* = 0.0001, *R*^2^ = 0.00001, *P* = 0.980), indicating that invasive or native status was not confounded with the geographic distances between individual plots. Across all watersheds we found that AM fungal community composition did not differ by status of vegetation (Permanova, *Pseudo*-*F* = 0.929, *R*^2^ = 0.055, *P* = 0.591). Within watersheds we also found that AM fungal community composition did not differ by status of vegetation (Fig. 3a, native vs. invasive, Permanova, *Pseudo*-*F* = 0.929, *R*^2^ = 0.055, *P* = 0.587, strata = watersheds). Rather, AM fungal community composition differed among watersheds (Fig. 3a, Permanova, *Pseudo*-*F* = 1.757, *R*^2^ = 0.19, *P* = 0.0003).

Although we found more AM fungi OTUs in the invasive plots, indicator species analysis among all the 18 plots showed that none of the individual OTUs were statistically associated with the native or invasive vegetation. When considering the invasion status and watershed, OTU 25 (Glomeraceae clade I in this study) was found to be statistically associated with the invasive plots only in the Palikea watershed (*Statistic* = 1, *P* = 0.034). For the Pahole and Manuwai watersheds there were no indicator OTUs in either invasive or native plots. Indicator species analysis considering watershed, but not invasion status showed that OTU 10 (Glomeraceae clade II in this study) was significantly associated with Palikea (*Statistic* = 0.772, *P* = 0.042). No other OTUs were associated with the other two watersheds.

### Phylogenetic community structure

**Fig. 4:**
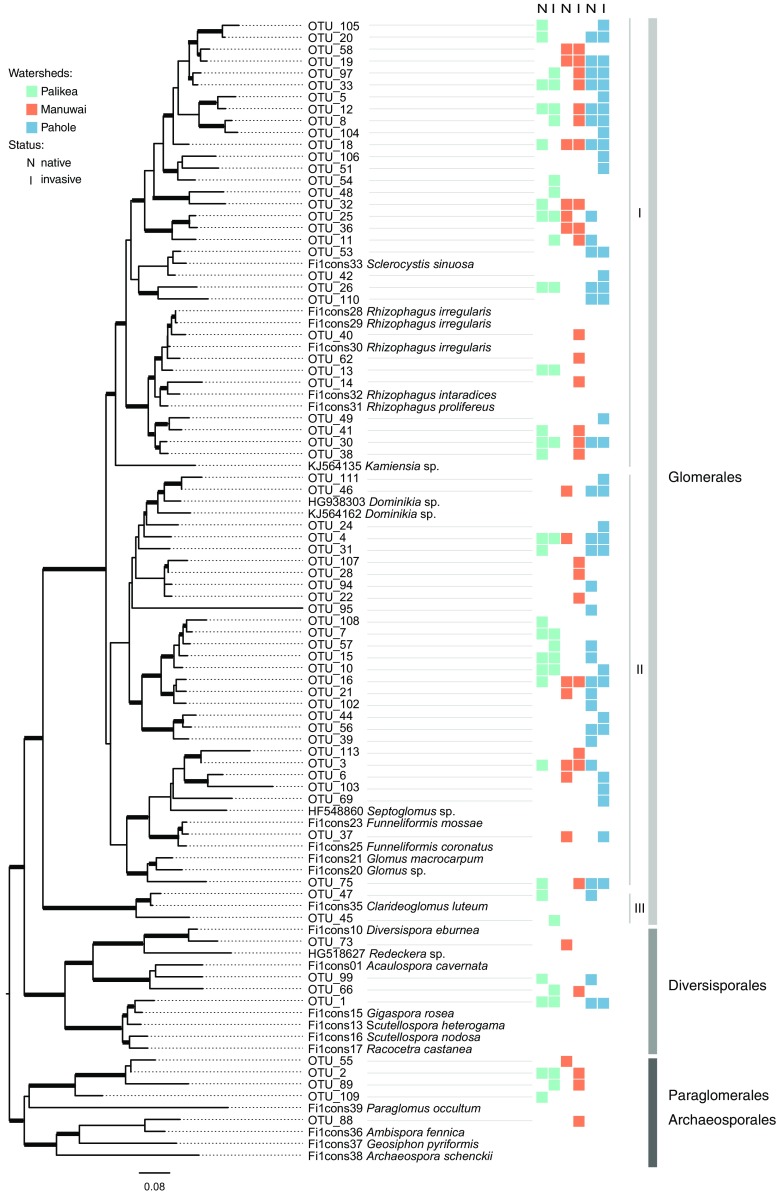
Phylogeny of the Glomeromycotina OTUs found in this study. Sequences with taxonomical identification correspond to curated sequences of Glomeromycotina (Öpik et al. 2010; Krüger et al. 2012) used as a backbone tree to facilitate the alignment of the short LSU sequences originated in this study. Squares indicate the presence of each OTU among the three watersheds in the native and invasive dominated plots

Based on the phylogenetic reconstruction of the 70 AM fungi OTUs, we found that they belong to three orders, seven families and at least 11 genera of Glomeromycotina fungi (Fig. 4). Similar to our AM fungi OTU incidence results, but considering only the phylogenetic community distances given by *comdist*, AM fungal community composition did not vary significantly by invasion status of the vegetation within watersheds (Fig. 3b, Permanova, *Pseudo*-*F* = 0.937, R^2^ = 0.055, *P* = 0.794, strata = watersheds), but differed significantly among watersheds (Fig. 3b, Permanova, *Pseudo*-*F* = 1.198, R^2^ = 0.14, *P* = 0.002). Using both metrics (OTU incidence and phylogenetic diversity) AM fungal communities varied significantly among watersheds regardless of invasion status (see Permanova results above), however each metric suggests a different pattern of community composition among watersheds (Mantel test *r* = 0.138, *P* = 0.221, Fig. 3).

Similar patterns were retrieved when testing within individual clades of AM fungi. There were no significant differences in AM fungal community structure based on invasion status of the vegetation for all the clades (Table 1). However, all clades were significantly stratified among watersheds except clade I of Glomeraceae which was present in all plots across all watersheds (Table 1). According to our phylogeny, this clade is composed of AM fungi belonging to the genera *Rhizophagus*, *Sclerocystis* and *Kamiensia* (Fig. 4).

**Table 1:** Structure of fungal community composition considering individual clades within Glomeromycotina (see Fig. 3) according to Permanova

Clade	Invasion status		Watershed	
	*R* ^2^	*P* value	*R* ^2^	*P* value
Glomerales	0.055	0.677	0.137	0.044*
Glomeraceae	0.055	0.680	0.138	0.040*
Glomeraceae clade I	0.051	0.696	0.121	0.359
Glomeraceae clade II	0.036	0.917	0.281	0.002**

As indicated by the “invasion status” column there are no significant differences in the phylogenetic structure of AM fungi among native and invasive plots. Except for the Glomeraceae clade I, all the other Glomeromycotina clades are structured according to watershed

### Soil chemistry and environmental data

Soil phosphorous, potassium, calcium and magnesium were statistically indistinguishable among the three watersheds, while elevation, mean precipitation and soil nitrogen varied significantly by watershed (Table 2). However, the RDA model that incorporated AM fungal community composition and environmental variables was not significant (RDA; *df* = 8, F = 1.1679, *P* = 0.422), suggesting that environment did not strongly influence AM fungal community composition among watersheds (Figure S3). Furthermore, according to invasion status of the vegetation, there were no significant differences in elevation, mean precipitation and soil chemistry across watersheds (Table 2), indicating that changes in AM fungal diversity due to invasion are not confounded with environment.

**Table 2:** Statistical results of Kruskal–Wallis test (Chi squared) and Mann–Whitney–Wilcoxon rank sum test (W) with Bonferroni corrections for comparisons of elevation, precipitation and soil chemistry composition among the three watersheds (Chi squared), and between native and invaded plots (W), *P* values of ≤ 0.05 are considered significant

	Chi squared	*P* value	W	*P* value
Elevation	14.363	0.002	41.0	1.000
Precipitation	14.452	0.002	38.5	0.890
pH	6.763	0.102	50.5	0.401
P	4.345	0.342	44.0	0.791
K	2.543	0.841	60.0	0.093
Ca	5.696	0.174	51.0	0.390
Mg	2.608	0.814	21.0	0.094
N	8.714	0.038	37.5	0.830

## Discussion

The successful establishment of species to new environments is a complex process that depends on innumerous factors. For plants with high mycorrhizal dependency, success in new areas is linked to the success of their mutualistic partners. However, in addition to partner compatibility, there are several abiotic and biotic filters underlying the establishment of mycorrhizal plants and fungi (Gladieux et al. 2015). We studied the impact of tree-dominated plant invasions on the richness, community composition and phylogenetic structure of AM fungi in Hawaiian soils. Despite the different compositions of native and invasive plant species among plots, we found that invasions consistently increased the richness of AM fungi. Furthermore, we found no indication that AM fungal community composition or richness was related to any of the environmental factors we considered. Rather, AM fungal community composition was stratified by geographic location, in this case, watersheds. This finding was consistent whether we took into consideration the phylogenetic structure of AM fungal communities or not.

This study provides new data on the effects of biological invasions in understudied tropical ecosystems that experience inordinate negative effects of invasions. Though we are far from finding any axioms for the effects of plant invasions on mycorrhizal communities, by adding new data from tropics, our results complement those of Lekberg et al. (2013). Based on OTU incidence data, Lekberg et al. (2013) found an increase in AM fungal diversity with plant invasions in alpine habitats. They suggest that resource availability and the ability of hosts to supply carbon to AM fungi may be an important driver of AM fungal diversity in their study system. Host supply of resources may also be a key factor affecting AM fungal richness in our system where invasive hosts are known to have greater rates of resource acquisition and often higher demands for resources than natives, especially for growth-limiting elements such as water and light (Cavaleri et al. 2014; Kagawa et al. 2009; Durand and Goldstein 2001).

An additional factor leading to an increase in AM fungal richness among invasive plots could be co-invasions of AM fungi with introduced hosts. However, based on our indicator analyses we did not detect clear differences between the AM fungi present in native or invasive plots. The lack of detectable differences in AM fungal community composition among native and invasive sites suggests that if any AM fungi are co-introduced, they are not restricted to sites dominated by non-native plants. While we found little evidence that aboveground invasions systematically reduced or increased the presence of any particular AM fungi OTUs, we did find that subsets of our AM fungi OTUs were strictly found in either native or invasive sites. In these cases, host identity is likely playing a crucial role in determining AM fungal communities.

Besides direct host-symbiont interactions there may be indirect effects of other invasive species on AM fungal diversity especially in our invaded plots. There, higher AM fungal diversity may in part be due to the presence of invasive ungulates and other invasive mammals such as rodents that have continually been moving soil around within these areas, but have been excluded from native habitats for greater than a decade. Within native areas, this lack of soil mixing may have led to more heterogeneous AM fungal communities (Wood et al. 2015). However, since non-native herbivores often reduce, rather than increase mycorrhizal diversity (Rossow et al. 1997) and both native and invasive plots have a similar long-term histories of invasive mammal activity, this effect seems less likely to have led to the increase in AM fungal richness that we observed in the invasive plots. Furthermore, even if invasive mammals were the primary driver of differences in AM fungal richness among habitat types, this is still evidence that biological invasions can have non-neutral and indirect effects on microbial mutualist communities.

Based both on OTU incidence and phylogenetic inference we found significant differences in AM fungal community composition among watersheds that were not related to environmental factors or vegetation type. These findings support the idea that rather than invasion status of the vegetation, geographic features such as high ridge tops may be effective barriers for the migration of most AM fungi among watersheds. However, had we targeted roots of specific native and non-native hosts, rather than bulk soil for our analyses, we may have found that invasive hosts versus native ones harbored discrete AM fungal communities. Thus the lack of observed differences in AM fungal community membership within watersheds may be owed to AM fungi dispersing within these geographic boundaries, which is independent of whether they are actively colonizing a host or not.

We found that all clades of AM fungi were stratified among watersheds except for clade I of Glomeraceae (Fig. 4), which was present among all three watersheds. This clade contains some of the most globally widespread and common AM fungal taxa and some of the few taxa to disperse well by air (Moora et al. 2011; Kivlin et al. 2011; Egan et al. 2014; Davison et al. 2015). The fact that these taxa are found throughout our study sites, and that they have arrived and established in Hawaii (the most remote oceanic island archipelago on Earth), lends additional support to their cosmopolitan nature. This result also indicates that the dispersal biology of AM fungi clades likely differ. Dispersal ability of AM fungi has been shown to vary based on species identity (Egan et al. 2014), and it would be interesting to test if dispersal traits are conserved at deeper phylogenetic levels. The abundance of Glomeraceae clade I across our study sites and the common pattern of long-tail species (OTU) frequency distributions of environmental microbial communities (Shoemaker et al. 2017), also found here (SI Figure S4) helps to explain why the *q* = 2 confidence intervals overlap (Fig. 2c). This estimator places more weight on the frequency of abundant species and discounts rare ones (Chao et al. 2014), thus it is the least likely of the three Hill numbers and estimators to accurately represent our true OTU diversity.

We found that despite significant differences among watersheds in soil nitrogen, elevation and precipitation there is no evidence that the community composition of AM fungi is related to these, or other environmental factors. This finding is in contrast to previous studies where environmental conditions such as temperature and pH were strong predictors of AM fungal diversity (Dumbrell et al. 2010; Davison et al. 2015). This result is surprising given that Hawaii is renowned for its strong environmental gradients that have led to multiple adaptive radiations (e.g. Baldwin and Sanderson 1998; Gillespie 2004; Tokita et al. 2017). In addition to the relatively greater potential importance of host and geography rather than local environment in determining AM fungal community composition, it is also possible that our sampling sites did not represent strong enough environmental gradients to see a response in AM fungal community composition, that our target locus for sequencing is not variable enough to detect these differences (Bruns and Taylor 2016), or that we did not measure the environmental factors of import for determining AM fungal community membership.

It is important to highlight that there is no universal way to study AM fungal communities (Hart et al. 2015). It is possible that the same experimental set up followed by different methods would yield different outcomes. Hart et al. (2015) show that factors ranging from preservation methods of soil samples, to choice of genetic marker and subsequent bioinformatics, may influence the results of every study. Both preservation methods and genetic marker choice may lead to biases towards specific AM fungal groups, which makes the comparison across studies challenging. Sample preservation is a crucial step in preserving DNA of AM fungi. Although snap-freezing in liquid nitrogen is probably the most efficient preservation method, it is not possible to use this method in many circumstances, namely when sampling in remote places. In our study, we used oven drying (50–60 °C; Janoušková et al. 2015), which has been shown to be efficient at preserving DNA of AM fungi while being the least expensive and simplest preservation method. Also, it has been suggested as one of the best preservation methods of AM fungal DNA (Hart et al. 2015). As for our choice of genetic marker, the LSU primers used in this study are known to select against certain AM fungal taxa in the Paraglomerales, Archaeosporales and Diversisporales (Krüger et al. 2009). Despite these overarching biases inherent to each specific method, all samples from this study were handled the same. Thus, while we acknowledge that we may not have assessed AM fungal diversity and community composition in their entireties, any methodological bias was equal across all our samples, which makes relative comparisons valid. Furthermore, because we used practices common in other studies of AM fungi our results are extractable and comparable to prior, and future studies.

Overall, our results suggest that aboveground invasions can lead to an increase in belowground microbial symbiont richness, but not changes in community membership, and that particular environmental conditions do not always lead to the assembly of certain taxa. Our results also support the diffuse nature of the arbuscular mycorrhizal symbiosis, even under biological invasions. We posit that factors such as host identity and functional traits as well as AM fungal dispersal barriers may play important roles in determining mycorrhizal diversity and deserve further attention. To disentangle the contribution of each to AM fungal community dynamics, more exhaustive studies need to be carried out. Future research that investigates the mycorrhizal community dynamics of invasive plant species in their native habitats relative to their introduced ranges, and are aimed at understanding the mechanisms driving AM fungi species coexistence would be particularly valuable. Lastly, mycorrhizal surveys should be designed to consider temporal and spatial effects, because time since invasion and the geographic scale at which observations of biodiversity are made can affect the perceived impact of biological invasions (Fridley et al. 2007; Powell et al. 2013; Chase et al. 2015). Such integrative approaches are necessary to shed additional light on the causal factors and consequences of biological invasions on microbial symbiont communities and their hosts.

## Electronic supplementary material

Below is the link to the electronic supplementary material.

Supplementary material 1 (DOC 681 kb)
