# Supplementary material for: Biological invasions increase the richness of arbuscular mycorrhizal fungi from a Hawaiian subtropical ecosystem
Source: Biol Invasions. 2018 Mar 21;20(9):2421–37. doi: 10.1007/s10530-018-1710-7 (PMC6417436; doi:10.1007/s10530-018-1710-7)
Supplement: Supplementary file 1 — Supplementary material 1 (DOC 681 kb) [file 10530_2018_1710_MOESM1_ESM.doc]

**Fig. S1** Diagram of our sampling plots. The plot is 24m × 24m; parallel gridlines are separated by 2m. Circles show soil core sampling locations. Color bands show the six size-classes into which soil cores were pooled following extraction. Blue X’s show locations where additional soil cores were sampled for soil chemical data.


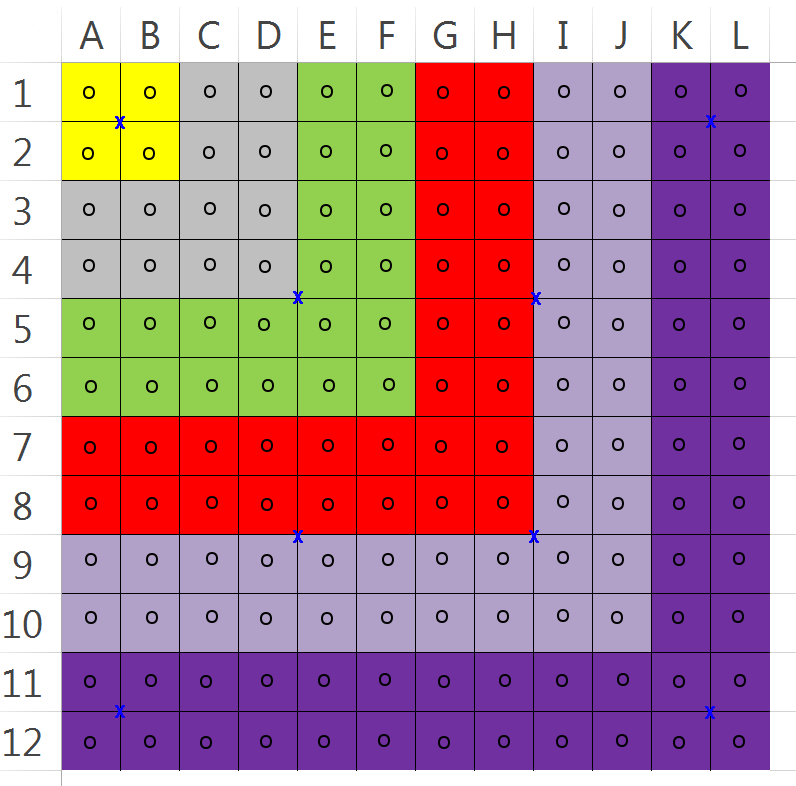


**Fig. S2** Rarefaction curves obtained for the fungal OTUs for the total 18 plots in the three watersheds (Palikea, Manuwai and Pahole) dominated by native (full lines) and invasive (dashed lines) vegetation.

**Fig. S3** Partial redundancy analysis (RDA) plot on the effect of environmental variables (elevation, soil nitrogen, potassium, phosphorus, pH, calcium, magnesium and annual mean precipitation, controlling for geographic location and watershed) on AMF community composition in the three watersheds (K: Palikea, M: Manuwai, P: Pahole) and by vegetation status (N: native, I: invasive).

**Fig. S4** OTU frequency distribution based on number of reads per OTU.

**Table S1** Geographic, climactic and edaphic metadata for each of our sampled plots. Rainfall information is taken from Giambelluca et al. (2012). Nutrient analyses were performed by the University of Hawaii’s Center for Tropical Agriculture and Human Resources following the methods of Carter & Gregorich (2008).

| Plot | Watershed | Invaded/Native-dominated | Replicate number | Latitude | Longitude | Altitude (M) | pH | P (mg/kg) | K (mg/kg) | Ca (mg/kg) | Mg (mg/kg) | N (mg/kg) | Mean annual rainfall (mm) |
| --- | --- | --- | --- | --- | --- | --- | --- | --- | --- | --- | --- | --- | --- |
| M1N | Manuwai | Native-dominated | 1 | 21.523 | -158.126 | 478 | 5.5 | 31 | 152 | 5402 | 1691 | 1.2 | 1300.6 |
| M2N | Manuwai | Native-dominated | 2 | 21.522 | -158.126 | 458 | 6.4 | 30 | 152 | 7639 | 1868 | 0.67 | 1338.8 |
| M3N | Manuwai | Native-dominated | 3 | 21.52 | -158.124 | 570 | 4.7 | 17 | 109 | 364 | 2811 | 0.73 | 1307.1 |
| M1I | Manuwai | Invaded | 1 | 21.523 | -158.124 | 499 | 5.6 | 22 | 533 | 2989 | 251 | 0.48 | 1271 |
| M2I | Manuwai | Invaded | 2 | 21.523 | -158.125 | 518 | 5.4 | 27 | 159 | 2991 | 1042 | 0.9 | 1300.6 |
| M3I | Manuwai | Invaded | 3 | 21.521 | -158.126 | 480 | 5.5 | 31 | 224 | 5820 | 1730 | 1.24 | 1338.8 |
| P1N | Pahole/Kahanahaiki | Native-dominated | 1 | 21.543 | -158.191 | 515 | 6.3 | 215 | 772 | 4166 | 1986 | 0.43 | 1334.4 |
| P2N | Pahole/Kahanahaiki | Native-dominated | 2 | 21.537 | -158.194 | 671 | 4.8 | 8.9 | 79 | 1002 | 1259 | 0.51 | 1384.8 |
| P3N | Pahole/Kahanahaiki | Native-dominated | 3 | 21.536 | -158.194 | 702 | 4.9 | 18 | 139 | 1566 | 201 | 0.71 | 1384.8 |
| P1I | Pahole/Kahanahaiki | Invaded | 1 | 21.54 | -158.194 | 619 | 5.3 | 105 | 639 | 2365 | 415 | 0.65 | 1367.3 |
| P2I | Pahole/Kahanahaiki | Invaded | 2 | 21.538 | -158.194 | 664 | 5.3 | 17 | 387 | 1934 | 1048 | 0.63 | 1367.3 |
| P3I | Pahole/Kahanahaiki | Invaded | 3 | 21.541 | -158.192 | 581 | 6 | 78 | 979 | 3906 | 642 | 0.68 | 1357.8 |
| K1N | Palikea | Native-dominated | 1 | 21.41 | -158.097 | 834 | 4 | 37 | 296 | 282 | 1426 | 0.95 | 1148.7 |
| K2N | Palikea | Native-dominated | 2 | 21.416 | -158.099 | 897 | 4.2 | 83 | 296 | 760 | 363 | 1.71 | 1165.7 |
| K3N | Palikea | Native-dominated | 3 | 21.415 | -158.097 | 816 | 5.2 | 71 | 450 | 2311 | 1087 | 1.24 | 1158.4 |
| K1I | Palikea | Invaded | 1 | 21.409 | -158.098 | 814 | 5.1 | 32 | 179 | 1821 | 912 | 1.01 | 1150.8 |
| K2I | Palikea | Invaded | 2 | 21.409 | -158.099 | 845 | 5 | 44 | 213 | 802 | 729 | 0.69 | 1150.8 |
| K3I | Palikea | Invaded | 3 | 21.416 | -158.099 | 887 | 4.5 | 55 | 760 | 2336 | 408 | 1.57 | 1165.7 |

**Methods S1:** Detailed results of Permanova analysis per section in the Results.

**Estimates of AMF diversity in native and invasive plots**

**Spatial aggregation ~ Status**

Permutation: free

Number of permutations: 1000

Terms added sequentially (first to last)

Df SumsOfSqs MeanSqs F.Model R2 Pr(>F)

Status 1 8114 8114 0.00012827 0.00001 0.98

Residuals 16 1012109875 63256867 0.99999

Total 17 1012117989 1.00000

**Plot ~ Status**

Permutation: free

Number of permutations: 1000

Terms added sequentially (first to last)

Df SumsOfSqs MeanSqs F.Model R2 Pr(>F)

Status 1 0.3024 0.30242 0.9048 0.05352 0.5814

Residuals 16 5.3479 0.33424 0.94648

Total 17 5.6503 1.00000

**Plot ~ Status, strata = watershed**

Blocks: strata

Permutation: free

Number of permutations: 1000

Terms added sequentially (first to last)

Df SumsOfSqs MeanSqs F.Model R2 Pr(>F)

Status 1 0.3024 0.30242 0.9048 0.05352 0.4146

Residuals 16 5.3479 0.33424 0.94648

Total 17 5.6503 1.00000

**Plot ~ Watershed, strata = status**

Blocks: strata

Permutation: free

Number of permutations: 1000

Terms added sequentially (first to last)

Df SumsOfSqs MeanSqs F.Model R2 Pr(>F)

Watershed 2 1.3354 0.66772 2.3212 0.23635 0.000999 ***

Residuals 15 4.3149 .28766 0.76365

Total 17 5.6503 1.00000

---

Signif. codes: 0 '***' 0.001 '**' 0.01 '*' 0.05 '.' 0.1 ' ' 1

**Phylogenetic community structure**

**Comdist ~ watershed**

Permutation: free

Number of permutations: 1000

Terms added sequentially (first to last)

Df SumsOfSqs MeanSqs F.Model R2 Pr(>F)

Watershed 2 0.29251 0.14625 1.1975 0.13768 0.002997 **

Residuals 15 1.83200 0.12213 0.86232

Total 17 2.12450 1.00000

---

Signif. codes: 0 '***' 0.001 '**' 0.01 '*' 0.05 '.' 0.1 ' ' 1

**Comdist ~ Status**

Blocks: strata

Permutation: free

Number of permutations: 1000

Terms added sequentially (first to last)

Df SumsOfSqs MeanSqs F.Model R2 Pr(>F)

Status 1 0.11749 0.11749 0.93663 0.0553 0.6823

Residuals 16 2.00701 0.12544 0.9447

Total 17 2.12450 1.0000

**Table 1:**

**Glomerales ~ Status**

adonis(formula = comm_Glom.mpd.dist ~ Status, data = metadata, permutations = 1000)

Permutation: free

Number of permutations: 1000

Terms added sequentially (first to last)

Df SumsOfSqs MeanSqs F.Model R2 Pr(>F)

Status 1 76522 76522 0.93866 0.05542 0.6773

Residuals 16 1304374 81523 0.94458

Total 17 1380897 1.00000

**Glomerales ~ Watershed**

adonis(formula = comm_Glom.mpd.dist ~ Watershed, data = metadata, permutations = 1000)

Permutation: free

Number of permutations: 1000

Terms added sequentially (first to last)

Df SumsOfSqs MeanSqs F.Model R2 Pr(>F)

Watershed 2 188924 94462 1.1887 0.13681 0.04396 *

Residuals 15 1191973 79465 0.86319

Total 17 1380897 1.00000

---

Signif. codes: 0 '***' 0.001 '**' 0.01 '*' 0.05 '.' 0.1 ' ' 1

**Glomeraceae ~ Status**

adonis(formula = comm_Glomeraceae.mpd.dist ~ Status, data = metadata, permutations = 1000)

Permutation: free

Number of permutations: 1000

Terms added sequentially (first to last)

Df SumsOfSqs MeanSqs F.Model R2 Pr(>F)

Status 1 72925 72925 0.92991 0.05493 0.6803

Residuals 16 1254748 78422 0.94507

Total 17 1327673 1.00000

**Glomeraceae ~ Watershed**

adonis(formula = comm_Glomeraceae.mpd.dist ~ Watershed, data = metadata, permutations = 1000)

Permutation: free

Number of permutations: 1000

Terms added sequentially (first to last)

Df SumsOfSqs MeanSqs F.Model R2 Pr(>F)

Watershed 2 183314 91657 1.2014 0.13807 0.04096 *

Residuals 15 1144359 76291 0.86193

Total 17 1327673 1.00000

---

Signif. codes: 0 '***' 0.001 '**' 0.01 '*' 0.05 '.' 0.1 ' ' 1

**Glomeraceae clade 1 ~ Status**

adonis(formula = comm_Glomeraceae_clade1.mpd.dist ~ Status, data = metadata, permutations = 1000)

Permutation: free

Number of permutations: 1000

Terms added sequentially (first to last)

Df SumsOfSqs MeanSqs F.Model R2 Pr(>F)

Status 1 24229 24229 0.8586 0.05093 0.6963

Residuals 16 451499 28219 0.94907

Total 17 475727 1.00000

**Glomeraceae clade I ~ Watershed**

adonis(formula = comm_Glomeraceae_clade1.mpd.dist ~ Watershed, data = metadata, permutations = 1000)

Permutation: free

Number of permutations: 1000

Terms added sequentially (first to last)

Df SumsOfSqs MeanSqs F.Model R2 Pr(>F)

Watershed 2 57794 28897 1.0371 0.12149 0.3586

Residuals 15 417934 27862 0.87851

Total 17 475727 1.00000

**Glomeraceae clade II ~ Status**

adonis(formula = comm_Glomeraceae_clade2.mpd.dist ~ Status, data = metadata, permutations = 1000)

Permutation: free

Number of permutations: 1000

Terms added sequentially (first to last)

Df SumsOfSqs MeanSqs F.Model R2 Pr(>F)

Status 1 34173 34173 0.59246 0.03571 0.9171

Residuals 16 922877 57680 0.96429

Total 17 957050 1.00000

**Glomeraceae clade 2 ~ Watershed**

adonis(formula = comm_Glomeraceae_clade2.mpd.dist ~ Watershed, data = metadata, permutations = 1000)

Permutation: free

Number of permutations: 1000

Terms added sequentially (first to last)

Df SumsOfSqs MeanSqs F.Model R2 Pr(>F)

Watershed 2 268863 134431 2.9301 0.28093 0.001998 **

Residuals 15 688187 45879 0.71907

Total 17 957050 1.00000

---

Signif. codes: 0 '***' 0.001 '**' 0.01 '*' 0.05 '.' 0.1 ' ' 1

**References:**

Carter, M.R. & Gregorich, E.G. ed. (2008) *Soil Sampling and Methods of Analysis*, second edition. CRC Press, Taylor & Francis, Boca Raton, Florida.

Giambelluca TW, Chen Q, Frazier AG, Price JP, Chen Y-L, Chu P-S, et al.*,* (2012) Online rainfall atlas of Hawai‘i. *Bull. Am. Meteorol. Soc.* **94**:313–316.
